# Established and Emerging Approaches for the Management of Dyslipidaemia

**DOI:** 10.6064/2012/482423

**Published:** 2012-09-10

**Authors:** Giuseppe Danilo Norata

**Affiliations:** ^1^Department of Pharmacological and Biomolecular Sciences, Università degli Studi di Milano 20122 Milan, Italy; ^2^Center for the Study of Atherosclerosis, Società Italiana Studio Aterosclerosi, Ospedale Bassini, 20092 Cinisello Balsamo, Italy; ^3^Centre for Diabetes, The Blizard Institute, Barts and The London School of Medicine and Dentistry, Queen Mary University, London E12AT, UK

## Abstract

The key role of dyslipidaemia in determining cardiovascular disease (CVD) has been proved beyond reasonable doubt, and therefore several dietary and pharmacological approaches have been developed. The discovery of statins has provided a very effective approach in reducing cardiovascular risk as documented by the results obtained in clinical trials and in clinical practice. The current efficacy of statins or other drugs, however, comes short of providing the benefit that could derive from a further reduction of LDL cholesterol (LDL-C) in high-risk and very high risk patients. Furthermore, experimental data clearly suggest that other lipoprotein classes beyond LDL play important roles in determining cardiovascular risk. For these reasons a number of new potential drugs are under development in this area. Aim of this review is to discuss the available and the future pharmacological strategies for the management of dyslipidemia.

## 1. Dyslipidaemia and Atherosclerosis

Atherosclerosis is a multifactorial disease in which plaque formation is the final process for several common pathogenetic mechanisms, including the individual susceptibility of genetic origin, hemodynamic stress, and various combinations of risk factors such as hypercholesterolemia, hypertension, diabetes, immune reactions and autoimmune diseases, inflammation, viral infections, and cigarette smoking [1–3].

The initiating event in atherosclerosis is the subendothelial retention of apolipoprotein apoB-containing lipoproteins in the arterial wall. This process is strictly related to the plasma levels of apoB-lipoproteins, however other properties can influence it, including lipoprotein size, charge and composition, and endothelial permeability. Very large lipoprotein (nonhydrolyzed chylomicrons) cannot enter the arterial wall, thus they do not directly promote atherosclerosis [4]; remnant chylomicrons, that are smaller, can enter the arterial wall, and can be retained [5]. LDL, which is the major cholesterol carrier in human plasma, has a key role in the initiation of the atherosclerotic process as confirmed by the great efficacy of LDL-lowering therapies in the prevention of cardiovascular disease [6–8]. Several factors may affect the endothelial permeability to lipoproteins, including the extent of the atherosclerotic lesion [9] and damages of the arterial wall. LDL concentration at the luminal surface may increase in areas where blood flow and shear stress are low and where the permeability of the endothelial layer is higher, thus increasing the entry of LDL in the intima in these sites [10].

The deposition and modification of LDL in the arterial wall promote a number of key processes including (1) impairment of endothelial function, (2) invasion of the arterial wall by leukocytes, particularly monocytes and T lymphocytes, (3) internalization of lipoproteins in macrophages and smooth muscle cells and accumulation of lipids, and (4) phenotypic modulation and proliferation of smooth muscle cells and synthesis of extracellular matrix.

Atherosclerotic lesions develop primarily in large and medium arteries, and above all in the intima, that is, the innermost layer of the arterial wall, consisting of a monolayer of endothelial cells adherent to a thin layer of connective tissue. The intima is separated from the tunica media, consisting of smooth muscle cells, collagen, and glycosaminoglycans, by the internal elastic lamina. 

The evolution of the atherosclerotic lesion is characterized by three stages [2].

1. Fatty streak formation [11]: the process begins with the accumulation of LDL (low-density lipoprotein) in the subendothelial space of the artery wall, where they interact specifically with components of the extracellular matrix [12, 13] and undergo several modifications (oxidation, glycation, aggregation, or formation of immune complexes) [14, 15], thus inducing endothelial cell activation. Following oxidation, the LDL is internalized by macrophages (derived from circulating monocytes recruited in the subendothelial space by chemokines produced by the activated endothelium), with subsequent accumulation of intracellular lipids and the formation of “foam cells.”
2. Fibrous plaque formation: at this stage the lesion is enriched in macrophages and proliferating smooth muscle cells; moreover, the formation of connective tissue and intracellular and extracellular accumulation of lipids are characteristic of this step.
3. Complicated lesions are the most advanced form of fibrous plaques. An important feature of complicated lesions is the formation of a lipid core, whose dimensions are related to the stability of atherosclerotic plaque [16]. The extracellular lipids are derived either from an increased influx of lipids not neutralised by internalization or removal by the cells, and from dead cells. The phenomenon of cell death, which can occur either by apoptosis or necrosis [17], is then related to the physical possibility of a rupture of the plaque, with consequent problems of coronary thrombosis.

## 2. Established Approaches for the Management of Dyslipidaemia

The key role of dyslipidaemias in determining cardiovascular disease (CVD) has been proved beyond reasonable doubt, and therefore several dietary and pharmacological approaches are used in the clinical practice for the management of dyslipidemia (Table 1) [18]. These include molecules and nutraceuticals which influence the absorption of dietary cholesterol or the synthesis of endogenous cholesterol, impact triglyceride and/or fatty acid handling, or increase HDL levels.

### 2.1. Dietary Supplements and Functional Foods

There are many functional foods and dietary supplements that are currently promoted as beneficial for people with dyslipidaemia or for reducing the risk of CVD. Some of these products have been shown to have potentially relevant functional effects but have not been tested in long-term clinical trials and should therefore be utilized only when the available evidence clearly supports their beneficial effects on plasma lipid values and their safety. Based on the available evidence, foods enriched with phytosterols (1-2 g/day) may be considered for individuals with elevated TC and LDL-C values in whom the total CV risk assessment does not justify the use of cholesterol-lowering drugs [19].

### 2.2. Bile Acid Sequestrants

Bile acid sequestrants are anion exchange resins that bind bile acids in the gastrointestinal tract. Bile acids are synthesized in the liver from cholesterol and released into the intestinal lumen; however most of the bile acid is returned to the liver from the terminal ileum via active absorption. The bile acid sequestrants are not systemically absorbed or altered by digestive enzymes. Therefore, the beneficial clinical effects are indirect. By binding the bile acids, the drugs prevent the entry of bile acid into the blood and thereby remove a large portion of the bile acids from the enterohepatic circulation. The decrease in bile acid returned to the liver leads to upregulation of key enzymes responsible for bile acid synthesis from cholesterol. The increase in cholesterol catabolism to bile acids results in a compensatory increase in hepatic LDLR activity, clearing LDL-C from the circulation and thus reducing LDL-C levels [20]. These agents also reduce glucose levels in hyperglycemic patients [21–23]; however, the mechanisms behind this reduction are not fully understood. 

Compared with the first-generation bile acid sequestrants (cholestyramine and colestipol), the second-generation bile acid sequestrant colesevelam hydrochloride (HCl) exhibit a greater binding capacity for bile acids. Therapy with colesevelam can lower LDL-cholesterol levels by 15–19% [24, 25]; colesevelam can also be safely combined with statin therapy in patients who would benefit from additional LDL-C lowering, resulting in LDL-C reductions of 42–48% [26, 27]. No major effect on HDL-C has been reported, while TG may increase in some predisposed patients.

### 2.3. Cholesterol Absorption Inhibitors

Ezetimibe is a lipid-lowering drug that inhibits intestinal uptake of dietary and biliary cholesterol by binding to the Niemann-Pick C1-like 1 protein, a sterol transporter [28]. By inhibiting cholesterol absorption at the level of the brush border of the intestine, ezetimibe reduces the amount of lipoprotein cholesterol circulated to the liver. In response to reduced cholesterol delivery, the liver reacts by upregulating LDLR, which in turn leads to increased clearance of LDL from the blood. Ezetimibe reduces LDL-C in hypercholesterolemic patients by 15–22% [29]; combined therapy with ezetimibe and a statin provides an incremental reduction in LDL-C levels of 15–20% [30, 31]. Ezetimibe also decreases triglycerides by up to 8% and raises HDL-C by 1–4%. The capacity of ezetimibe/simvastatin to reduce risk for cardiovascular events in patients with CAD is being studied in the IMPROVE-IT trial [32]. Furthermore the SHARP trial [33] showed that lipid-lowering therapy with ezetimibe in combination with simvastatin is safe and significantly reduces the incidence of major atherosclerotic events in high-risk patients with advanced chronic kidney disease.

Ezetimibe can be used as second-line therapy in association with statins when the therapeutic target is not achieved at maximal tolerated statin dose or in patients intolerant of statins or with contraindications to these drugs.

### 2.4. Statins

Several clinical trials have demonstrated that statins significantly reduce cardiovascular morbidity and mortality in both primary and secondary prevention [34–40]. Statins slow the progression or promote the regression of coronary atherosclerosis; statin treatment significantly reduces the carotid intima-media thickness (IMT), a surrogate marker of atherosclerosis [41].

Statins inhibit HMG-CoA reductase activity resulting in the inhibition the conversion of acetyl-coenzyme A and acetoacetyl-coenzyme A to mevalonate, a key step in cholesterol synthesis. This inhibition leads to a reduced synthesis of cholesterol in the liver and in an increased expression of hepatic low-density lipoprotein receptor (LDLR), thus reducing the concentration of circulating LDL-C and other apoB-containing lipoproteins including TG-rich particles. All statins induce modest elevations in HDL-C [42], with differences among statins [43]. Current available evidence suggests that the clinical benefit is largely independent of the type of statin but depends on the extent of LDL-C lowering; therefore, the type of statin used should reflect the degree of LDL-C reduction that is required to reach the target LDL-C in a given patient [37].

Besides, statins exhibit several pleiotropic beneficial effects that are independent of cholesterol lowering properties [44, 45].

- The inhibition of HMG-CoA reductase lead to the inhibition of isoprenoid intermediates synthesis, including farnesyl pyrophosphate (FPP) and geranylgeranyl pyrophosphate (GPP). Isoprenylation of proteins is involved in the activation of inflammatory pathways [46] and in the vascular remodeling present in disease state such as atherosclerosis and diabetes [47]; blocking this metabolic pathway protects against the progression of atherosclerosis.
- Statins reduce platelet activation and aggregability in both cholesterol-dependent and cholesterol-independent manner [48, 49]. Furthermore, statins decrease LDL-induced platelet aggregation [50, 51].
- Statins reduce the proatherogenic effects of OxLDL by several ways, including the downregulation of macrophage and endothelial scavenger receptors, thus reducing the uptake of OxLDL [52–55].
- Statins promote eNOS production and function in endothelial cells by increasing eNOS expression and activity, and by preventing the downregulation of eNOS expression and activity induced by OxLDL [56].
- Statins promote endothelial progenitor cell proliferation, migration, and cell survival [57–60].
- Statins reduce vascular smooth muscle cell migration and proliferation, two key steps of atherogenesis process [61, 62], while promoting vsmc apoptosis [63, 64].
- Statins reduce the inflammatory response by inhibiting the induction of major histocompatibility complex class II (MHC-II), involved in the activation of T lymphocytes and in the control of immune response [65] and by decreasing CD40 expression and function in vascular cells [66].
- Statins stabilize atherosclerotic plaque by lipid lowering [67] and by decreasing the expression of matrix metalloproteinases and tissue factor [68, 69].
- Statins decrease myocardial remodeling, by inhibiting some effects of angiotensin II (a major effector of the renin-angiotensin system), including cardiac fibroblast proliferation, collagen synthesis, and induction of cardiomyocyte proliferation [70, 71], providing a beneficial effect for heart failure.

### 2.5. Fibrates

Increased triglyceride levels are key features in certain conditions that lead to premature vascular disease, including type 2 diabetes mellitus, familial combined hyperlipidaemia. and familial hypoalphalipoproteinaemia. Elevated levels of TG are closely correlated with low HDL-cholesterol levels.

Fibrates are agonists of peroxisome proliferator-activated receptor-*α* (PPAR-*α*); by interacting with PPAR-*α*, fibrates recruit different cofactors and regulate the expression of several genes involved in cholesterol transport and lipid metabolism. As a consequence, fibrates enhance degradation of triglyceride-rich particles by activation of lipoprotein lipase and decrease hepatic very low density lipoprotein production [72], resulting in a significant reduction of TG levels (up to 50%); they induce the synthesis of apoA-I and apoA-II, leading to a modest increase of HDL Cholesterol levels (up to 10–15% in short-term studies and <5% in the long-term intervention trials) [73], and promote a shift in the LDL-C particle distribution towards larger, more buoyant particles which are less susceptible to oxidation and possess higher affinity for the LDL receptor [74, 75]. Due to their effects, fibrates are commonly used in subjects with significant hypertriglyceridaemia.

However, clinical trial data on the role of fibrates in cardiovascular prevention are conflicting. Fenofibrate did not significantly reduce the risk of the primary outcome of coronary events in the FIELD trial [76]. Total cardiovascular events resulted decreased, due to reduced nonfatal myocardial infarctions and revascularisations. Furthermore, a meta-analysis of 10 randomized placebo-controlled trials revealed that long-term therapy with fibrates significantly reduces the occurrence of nonfatal myocardial infarction but has no significant effect on other adverse cardiovascular outcomes [77]. Finally, the results of the ACCORD trial showed that the combination of fenofibrate and simvastatin does not reduce the risk of cardiovascular disease in patients with type 2 diabetes mellitus, as compared with statin monotherapy [78]; only subgroups of patients with dyslipidemia seem to benefit from fibrate therapy. Thus, the overall efficacy of fibrates on cardiovascular outcomes is much less robust than that of statins.

### 2.6. Omega-3 Fatty Acids

Omega-3 fatty acids [eicosapentaenoic acid (EPA) and docosahexaenoic acid (DHA)] are components of fish oil and the Mediterranean diet and their use is beneficial for the cardiovascular system [79–81]. Omega-3 fatty acids decrease serum levels of VLDL and triglycerides by several mechanisms [82].

Clinical studies have shown the beneficial effect of omega-3 fatty acids. The GISSI Prevenzione trial showed that the use of omega-3 fatty acids was associated to significant reductions in the risk for reinfarction and sudden death among patients who sustained an acute coronary syndrome prior to randomization [83]; in another study, the addition of EPA to statin therapy resulted in a 19% incremental reduction in major coronary events compared with statin monotherapy [84].

Not all trials have demonstrated a positive effect of omega-3 supplementation on cardiovascular disease [85, 86]; for example, low-dose supplementation with omega-3 fatty acids did not reduce the incidence of major cardiovascular events in patients who have had a myocardial infarction [87].

Clinical and mechanistical studies are required to define the benefits of omega-3 fatty acids in both primary and secondary prevention.

### 2.7. Nicotinic Acid

Nicotinic acid has broad lipid-modulating action, raising HDL-C in a dose-dependent manner by *∼*25%, and reducing both LDL-C by 15–18% and TG by 20–40%. Nicotinic acid is unique in lowering Lp(a) levels by up to 30% at this dose. It is therefore primarily used in subjects with low HDL-C levels as typical of mixed hyperlipidemia, HTG, or in FCH, but may also be used in subjects with insulin resistance (type 2 diabetes and metabolic syndrome). Nicotinic acid has multiple beneficial effects on serum lipids and lipoprotein. In fact, nicotinic acid induces hepatic production of apoA-I and HDL [88]; furthermore it inhibits HDL particle uptake and catabolism in the liver [89]. Nicotinic acid reduces hepatic VLDL and TG secretion by several mechanisms. It decreases the flux of fatty acid from adipose tissue to the liver (due to the inhibition of hormone-sensitive lipase activity) [90]; it inhibits TG formation in the liver (by inhibition of diacylglycerol acyltransferase); it increases apoB catabolism, resulting in VLDL-cholesterol and LDL-cholesterol reduction.

Nicotinic acid may be used in combination with statins as a therapy for combined hyperlipidemia. Nicotinic acid is currently used mostly as an extended release (ER) form. In patients with established CHD, the addition of extended-release (ER) niacin to statin therapy results in the stabilization of CIMT, in contrast to patients receiving statin monotherapy who experienced significant CIMT progression, despite having a mean baseline LDL-C of 90 mg/dL on statin monotherapy [91]. CIMT regression resulted highly correlated with the degree of HDL-Cholesterol increase [92, 93].

Niacin usage is limited by cutaneous flushing, a bothersome adverse effect. Flushing is the leading cause for discontinuation of therapy, estimated at 25–40% or more [94, 95]. Flushing is mediated by prostaglandin D2 (PGD2), a potent vasodilator. PGD2 binds to DP1 receptors in the skin. ER niacin is associated with a lower frequency, intensity, and duration of flushing than immediate release niacin [96–98]. Laropiprant is an antagonist of the DP1 receptor, inhibits cutaneous flushing and significantly improves the tolerability of niacin by over 50% [96, 97].

In a recently published trial, the addition of niacin to statin therapy did not induce an incremental benefit in patients with established cardiovascular disease, low levels of HDL-C at baseline and levels of LDL-C at target (<80 mg/dL) [98]. 

## 3. Emerging Approaches for the Management of Dyslipidaemia

The key role of dyslipidaemias in determining cardiovascular disease (CVD) has been proved beyond reasonable doubt; and the discovery of statins has provided a very effective approach in reducing cardiovascular risk as documented by the results obtained in clinical trials and in clinical practice. Research however is clearly suggesting that other lipoprotein classes beyond low-density lipoprotein (LDL) play important roles in determining cardiovascular risk and that the current efficacy of statins or other drugs comes short of providing the benefit that could derive from a further reduction of LDL cholesterol (LDL-C) in high-risk and very-high-risk patients. For these reasons a number of new potential drugs are under development in this area. 

### 3.1. Interfering with Lipoprotein Synthesis: ApoB Silencing, MTP Inhibitors

**Figure 1:**
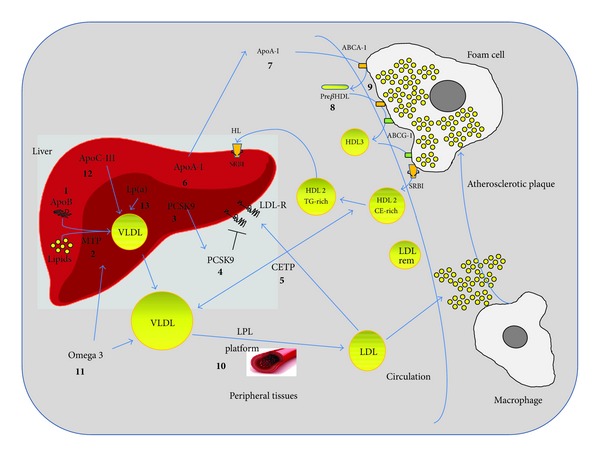
Emerging targets for dyslipidemia. The novel drugs that are under development for the treatment of dyslipidemia present several mechanisms of action. Emerging therapeutic agents for LDL lowering will: (a) interfere with lipoprotein synthesis in the liver by silencing apolipoprotein B (apoB) expression (1) or inhibiting microsomal triglyceride transfer protein (MTP) activity (2); (b) promote LDL-receptor activity by silencing (3) or blocking (4) proprotein convertase subtilisin/kexin type 9 (PCSK9). Emerging therapeutic agents affecting HDL will: (c) increase HDL-C plasma levels by blocking cholesteryl ester transfer protein (CETP) (5), or inducing apolipoprotein A-I (apoA-I) expression (6), (d) improve HDL activity by mimicking apoA-I (7) or nascent HDL (8) or increase the expression of receptors favoring cholesterol efflux from cells (9). Emerging therapeutic agents for triglycerides lowering will improve the catabolism of triglycerides and the handling fatty acids by peripheral organs (10), by new formulation of omega 3 fatty acids (11) and by inhibiting the expression of apolipoprotein C-III in the liver (12). Specific silencing of apolipoprotein (a) is also under investigation (13).

Hepatic biosynthesis of very-low-density lipoprotein (VLDL) is dependent on two dominant proteins, namely, apolipoprotein B (apoB) and microsomal triglyceride (TG) transfer protein (MTP). ApoB is an obligatory structural component of VLDL and requires progressive lipidation, mediated by the resident endoplasmic reticulum chaperone MTP, to maintain conformational integrity and folding during the process of lipoprotein assembly. Interfering with this process is therefore an attractive approach for reducing lipoprotein synthesis and decreasing plasma LDL-C concentration. The possibility of targeting apoB during the process of gene translation is under extensive investigation. One approach to block mRNA translation of a gene is through the use of a single-strand antisense oligonucleotide (ASO) that is complementary to the mRNA. Following hybridization to the mRNA, the ASO inhibits translation and splicing and leads to degradation of the mRNA by RNase [99]. ASO kinetics are characterized by a large and rapid distribution to the liver following parenteral administration, thus making this approach quite attractive for inhibiting mRNA after transcriptional processing. This results in a reduced synthesis of proteins in the liver, such as apoB [100] (Figure 1). Preclinical studies demonstrated that ASOs targeting apoB are quite effective in mice in reducing apoB mRNA liver levels in a dose-response manner [101] followed by a reduction in circulating LDL-C concentration, LDL particle number, circulating TG, and lipoprotein(a) [Lp(a)], while chylomicrons, which contain apoB-48, were spared, because of the high distribution of ASOs to the liver.

 Mipomersen is an ASO targeting apoB which leads to a dose-dependent reduction in apoB and total cholesterol [102] and was effective in phase II and phase III clinical studies in combination with statin therapy in individuals with LDL-C 100–220 mg/dL on a maximal tolerated statin dose with or without ezetimibe, bile-acid sequestrant and/or niacin and in patients with familial hypercholesterolaemia [102] as well as in monotherapy in individuals with mild-to-moderate hyperlipidaemia [103] and in high-risk statin-intolerant patients [104]. Overall, mipomersen provided significant further reduction in LDL-C (~30%) and other lipids when added to conventional lipid therapy. The most common adverse effects were injection-site reactions and flu-like symptoms. Liver fat accumulation was also observed in both phase II and phase III studies and is in line with the mechanism of action of the drug.

MTP, found in the endoplasmic reticulum of hepatocytes and enterocytes, mediates the formation of apoB-containing lipoproteins in the liver and in the intestine [105]. Mutations in the gene encoding MTP can cause abetalipoproteinemia, a rare genetic disease characterized by an absence of apoB-containing lipoproteins and severe malabsorption of fat and fat-soluble vitamins [105]. The genetic defect underlying abetalipoproteinemia suggests that inhibiting MTP may reduce circulating concentrations of cholesterol and apoB-containing lipoproteins (Figure 1). The MTP inhibitor lomitapide is currently in phase III testing. The drug, tested in monotherapy or in combination with conventional lipid-lowering therapy in homozygous FH [106] or in patients with hypercholesterolaemia (LDL-C 130–250 mg/dL) [107] showed a reduction in circulating LDL-C, apoB, total cholesterol, nonhigh-density lipoprotein cholesterol (non-HDL-C), and Lp(a) levels. Steatorrhea related to lomitapide treatment was effectively reduced by a fat-restricted diet; adverse effects such as elevated liver enzymes and hepatic-fat accumulation (expected from the mechanism of action) were reported and may restrict the patient population for this drug. However, for patients with homozygous FH that cannot be controlled with conventional lipid-lowering therapy, MTP inhibition may be a beneficial approach. Ongoing studies of lomitapide should provide additional information on the safety and tolerability of this agent and potential patient populations for whom it may be appropriate. 

### 3.2. Promoting LDL-Receptor Activity: PCSK9 Inhibitors

Cholesterol homeostasis is regulated by the LDL receptor (LDL-R) through its binding and uptake of circulating apoB-containing lipoproteins which are then internalized into the liver cell. The key mechanism associated with statins' action involves the increase of LDL-R expression on the hepatocyte surface, followed by increased LDL turnover and reduction of plasma cholesterol levels. This mechanism is partially dampened by a negative feedback response associated with the induction of the expression and secretion of proprotein convertase subtilisin/kexin type 9 (PCSK9) [108], a serine protease which promotes the degradation of LDL-R [109] thus attenuating, at least in part, lipid-lowering efficacy of statins and ezetimibe [110].

 Given that PCSK9 acts both intracellularly, as a chaperone directing the LDL-R to the lysosomes, and in the circulation, by promoting LDL-R internalization [110], the possibility of inhibiting PCSK9 represents a logical step to enhance the lipid-lowering effect of conventional agents [110] (Figure 1). To this end, at least five different human monoclonal antibodies and three gene-silencing approaches are under development. Among a series of antibodies against PCSK9, clinical trial results are available for two of them, SAR236553/REGN727 [111] and AMG145 [112], and these compounds are both in phase II or III development. A number of additional anti-PCSK9 monoclonal antibodies, in earlier clinical development, are currently being investigated for potential use in humans, including 1B20, PF-04950615/RN-316, and LGT 209.

To date the largest body of information is available for REGN727/SAR236553, a fully human monoclonal antibody, which binds to the catalytic domain of PCSK9 that interacts with LDL-R. Overall, results from phase I and II clinical trials suggest that s.c. injections of SAR236553/REGN727 dose dependently reduce PCSK9 activity and produce significant additional reductions in LDL-C as well as in non-HDL-C independently of statin treatment. The antibody was generally well tolerated over the treatment period, with no drug-related adverse effects on liver function tests or other laboratory parameters, and no serious treatment-emergent adverse effects [111, 113, 114]. The number of injection-site reactions (including erythema, pruritis, swelling, haematoma, and rash) was generally low and the few reported were mild in severity [113].

AMG145 is another fully human monoclonal antibody which also binds specifically to human PCSK9. Phase I data in subjects on stable statin therapy demonstrated a dose-dependent decrease in LDL-C and unbound PCSK9 with increasing subcutaneous doses of AMG145. LDL-C was lowered by up to 81% at maximal doses, over and above the LDL lowering achieved with statin alone [112]. Phase I data [112] indicated no serious adverse events in the AMG145 group compared with placebo, no discontinuations from the studies related to adverse events and only 1 case of transaminase elevation >3× upper limit of normal. Although the safety results for PCSK9 monoclonal antibodies are encouraging, it should be noted that the trials to date have been relatively short in duration and were conducted in relatively small patient populations. Further trials are therefore required to test the long-term safety and efficacy of PCSK9 monoclonal antibodies in larger and more varied patient populations. In this context, given that statin treatment increases PCSK9 levels, it should be considered that the frequency of injection should be increased accordingly in statin-treated patients for optimal PCSK9 inhibition.

PCSK9 can also be suppressed through gene silencing; among the nucleic acid-based therapies, the development of SPC5001, a locked nucleic acid-based inhibitor, and that of BMS-844421, an antisense RNA therapy, were terminated during phase I clinical trials. ALN-PCS02, an RNA interference molecule, is being tested in an ongoing phase I study in healthy volunteers to evaluate the safety and tolerability of various doses. In interim data on 20 subjects, robust target protein knockdown was observed at the highest dose tested, with a mean 60% reduction in plasma PCSK9 levels 3–5 days after administration. In line with PCSK9 genetics, this type of knockdown entailed a mean 39% reduction in LDL-C., with no drug-related discontinuations or liver enzyme elevations (http://www.clinicaltrials.gov/ct2/show/NCT01437059).

### 3.3. Increasing Plasma HDL-C Levels: CETP Inhibitors, ApoA-I Inducers

High-density lipoproteins (HDL) possess several physiological activities that may explain their antiatherosclerotic properties; among them, the most relevant is the ability of HDL to promote the efflux of excess cholesterol from peripheral tissues to the liver for excretion [115, 116]. Furthermore, apolipoproteins, lipids, and enzymes carried by HDL may perform additional antiatherosclerotic activities [117–119].

 In recent years, the metabolic pathways associated with HDL have been extensively investigated and elucidated, allowing the design of drugs able to interfere with HDL catabolism, improve the expression of the main protein constituent, namely, apoA-I, or mimic their activity.

 The pharmacological approaches under development can be grouped in two major clusters: molecules increasing plasma HDL levels and molecule improving HDL function. It is expected that an increase in HDL levels can be beneficial when associated with an improvement in HDL function.

 Recently, a mendelian randomization analysis revealed that a single nucleotide polymorphism in the endothelial lipase gene (LIPG Asn396Ser) associated with increased HDL-C levels in the population did not decrease the risk of myocardial infarction, despite a 13% reduction expected from the increased HDL-C levels [120]. Similarly, a genetic score combining 14 variants exclusively related to HDL-C was not associated with myocardial infarction risk [120], further challenging the concept that higher HDL-C levels will automatically translate into lower cardiovascular risk. In spite of these observations, clinical trials are still ongoing with drugs affecting HDL levels.

Cholesteryl ester transfer protein (CETP) is an enzyme involved in the transfer of cholesteryl esters from HDL to LDL and VLDL; this process results in a reduction and remodeling of HDL particles and in an increase of LDL and VLDL levels. Furthermore, CETP transfers TG from VLDL or LDL to HDL, resulting in the formation of TG-enriched HDL, which is easily hydrolyzed by hepatic lipase leading to TG-rich small HDL that are cleared more rapidly from the circulation [121]. Under pathological conditions, including atherosclerosis, CETP activity is increased; moreover, in humans, CETP deficiency results in increased HDL levels. Altogether these observations led to the concept that CETP inhibition is a powerful tool to increase HDL-C, decrease LDL-C and VLDL-C, and reduce the development of atherosclerosis [122].

 The first CETP inhibitor developed, torcetrapib, despite a 72% increase in HDL-C levels, was withdrawn because of an increased risk of cardiovascular events and death from any cause in the investigation of lipid levels management to understand its impact in atherosclerotic events (ILLUMINATE) trial [123]. Retrospectively, this effect was attributed to an off-target effect of torcetrapib such as the rising of systolic blood pressure by an average 5.4 mmHg [124], an effect associated with the stimulation of aldosterone synthesis via pathways independent of CETP inhibition [123, 125]. The possibility that CETP inhibition *per se* could generate larger cholesterol-enriched HDL with impaired cholesterol efflux potential was also proposed [124]. However this was not confirmed by *in vitro* studies. Among the three newer compounds, dalcetrapib, anacetrapib, and evacetrapib, with different potency toward CETP inhibition (evacetrapib > anacetrapib > dalcetrapib) and apparently lacking the off-target effects of torcetrapib, two remain under development, while that of dalcetrapib was recently halted.

 The decision to stop dalcetrapib was based on the dal-OUTCOMES trial interim analysis which showed that dalcetrapib, in acute coronary syndrome patients, failed to demonstrate a significant reduction in cardiovascular adverse events (http://www.roche.com/media/media_releases/med-cor-2012-05-07.htm). In contrast to the earlier CETP inhibitor, torcetrapib, no safety concerns were reported. In addition, the dal-VESSEL study showed that dalcetrapib reduced CETP activity and increased HDL-C levels without affecting nitric oxide-dependent endothelial function, blood pressure, or markers of inflammation and oxidative stress [126] while the dal-PLAQUE study demonstrated some beneficial vascular effects of the drug, including the reduction in total vessel enlargement over 24 months [127].

 While disappointing, the pursuit of an extensive programme of clinical trials and basic research to develop dalcetrapib has provided new information on the biology of HDL in both man and animal models, and on CETP inhibition as a viable therapeutic target for raising levels of HDL-C. Several other CETP inhibitors that raise HDL-C levels to a greater extent than dalcetrapib and also significantly lower LDLC and other novel HDL-raising agents remain under development. Ultimately, the benefits of each of these novel CETP inhibitors must be determined through prospective, randomized, clinical outcome trials. The possibility that, while CETP inhibitors were developed on the premise that they would increase HDL-C more than any therapy currently available, the benefit may still be largely due to the incremental lowering of LDL-C observed with the more potent inhibitors, should be considered for the transfer of these drugs in the clinical practice. 

The life cycle of HDL starts from lipid-poor apoA-I, termed nascent, or pre*β*-HDL (the latter on the basis of the characteristic electrophoretic mobility), which promotes cholesterol mobilization from the cell membrane mainly through the activation of ABCA-1. Pre-*β* HDL accumulates effluxed cholesterol and matures to *α* HDL, which further promotes cholesterol efflux via the activation of different transporters including ATP-binding cassette subfamily G member 1 protein (ABCG-1) and scavenger receptor class B member 1 (SR-BI) (Figure 1). Induction of the expression of apoA-I is therefore a cornerstone mechanism of drugs so far used to increase HDL levels, including fibrates and nicotinic acid [88]. A novel small synthetic molecule, RVX-208, is able to induce apoA-I synthesis and is under development. Preclinical studies in nonhuman primates showed the ability of this compound to increase plasma apoA-I and HDL-C levels by up to 60% and 97%, respectively. In humans, the ASSERT study showed that the administration of RVX-208 at a dose of 50, 100, or 150 mg twice daily for 12 weeks resulted in increases in apoA-I (up to 5.6%), HDL-C (by 3.2% to 8.3%), and large HDL particles (by 11.1% to 21.1%), although the primary endpoint of individual pairwise comparisons of apoA-I changes with placebo was not statistically significant [128]. These findings, although not at the level expected, require further evaluation, perhaps through the investigation of HDL functionality.

### 3.4. Improving HDL Activity

The rationale behind the development of HDL mimetics is the possibility of mimicking the first phase of the HDL life cycle and promoting cholesterol efflux, mainly from cholesterol-loaded cells in the vascular wall such as macrophages and foam cells (Figure 1).

 To this aim, lipid-poor apoA-I-phospholipid complexes have been extensively studied in preclinical models and preliminary studies in humans. So far, different approaches are under investigation. CSL-111 is a complex of native apoA-I and phosphatidylcholine isolated from soybeans which induced a significant reduction in atheroma volume compared with baseline [129]. The same study showed significantly reduced progression of coronary atherosclerosis in the CSL-111–treated group compared to placebo. Treatment with CSL-111, however, induced reversible alanine aminotransferase elevations exceeding 10 times the normal upper limit in one-third of patients receiving 80 mg of the compound, while no changes were observed in patients receiving 40 mg/kg [129]. To overcome this limitation, a reformulated version, CSL-112, with greater cholesterol efflux capacity and less hepatotoxicity, is in phase I study.

 A similar approach was tested also by incorporating recombinant apoA-I Milano, which differs from normal apoA-I by a cysteine-to-arginine substitution at amino acid 173. ETC-216 is a complex of apoA-I Milano with phospholipid and in a small clinical study significantly reduced total atheroma volume, measured by IVUS, in patients with acute coronary syndrome [130]. Since 2003, ETC-216 development was halted and only recently a different company bought the license and renamed the molecule MDCO-216 with the aim of starting larger clinical trials soon.

 CER-001, a synthetic recombinant human apoA-I HDL mimetic, is in phase II testing in approximately 500 patients with acute coronary syndrome, to determine the effect on atherosclerotic plaque progression/regression as assessed by IVUS (CHI SQUARE; http://clinicaltrials.gov/ct2/show/NCT01201837). Several other complexes of apoA-I and different phospholipids are in preclinical development and will soon enter clinical testing phases.

A second approach to improve HDL function is represented by small peptides design to mimic apoA-I function. The most well-studied of these peptides is 4F, consisting of 18 amino acids, which was designed to have the lipid-binding properties of apoA-I through a common secondary structure, the class A amphipathic helix. The use of D-amino acids (D-4F) enables oral delivery of this compound by conferring resistance to gastrointestinal proteolytic enzymes. Several preclinical studies showed that 4F promotes cholesterol efflux via ABCA1 and SR-BI, and possesses anti-inflammatory, antithrombotic and antioxidant properties. The only available human study of D-4F showed that HDL isolated from subjects treated with a single 300 mg or 500 mg dose of unformulated D-4F had increased inhibition of LDL-induced monocyte chemotaxis compared to HDL isolated from control subjects. Data on the safety profile of D-4F in humans are not available yet. Overall at least 22 apoA-I mimetics are under development [131]; however, with the exception of D-4F, the other peptides require parenteral administration and, in humans, data on efficacy, tolerability, and safety, including autoantibody generation, are lacking.

### 3.5. Dual PPAR Agonists

PPAR-*α* is highly expressed in liver and skeletal muscle, controls the genes involved in fatty acid oxidation, and plays a pivotal role in energy homeostasis and lipoprotein metabolism by inducing lipoprotein lipase and apoA-I expression. PPAR-*γ* is highly expressed in adipocytes, in addition to skeletal muscle, liver, and kidney, and has been shown to regulate the expression of genes that mediate adipocyte differentiation, energy metabolism, and insulin action. Therefore, a dual PPAR-*α* and -*γ* agonist may possess the beneficial effects of fibrates (PPAR-*α* agonists) on plasma lipids and thiazolidinediones (PPAR-*γ* agonists) on insulin sensitivity and potential anti-inflammatory effects. This approach could prove beneficial in effectively managing both glycaemic control and lipid profile particularly in patients with type 2 diabetes.

 Several attempts to develop a dual PPAR agonist for diabetes have so far failed because of various safety concerns: ragaglitazar, MK-0767, and naveglitazar were found to be associated with an increased incidence of bladder cancer and hyperplasia in rodent studies and tesaglitazar development was discontinued because of indications that it may cause kidney dysfunction.

 The dual agonist muraglitazar, a strong PPAR-*γ* agonist with moderate PPAR-*α* effects, effectively reduced haemoglobin A1c (HbA1c) and TG levels while increasing HDL-C levels [132]. The development of muraglitazar was stopped because of an excess incidence of the composite end point of death, major adverse cardiovascular events (myocardial infarction, stroke, and transient ischaemic attack), and congestive heart failure compared to placebo [133].

 The latest dual PPAR-*α*/*γ* agonist in development is aleglitazar, which is currently in phase III trials. This compound has a balanced affinity for both *α* and *γ* receptor subtypes. The phase II study SYNCHRONY has shown a significant dose-dependent reduction in HbA1c, in fasting plasma glucose, TG and LDL-C, and an increase in HDL-C [134]. Further analysis of this study indicated that aleglitazar produced a shift from atherogenic small dense LDL particles associated with type 2 diabetes to larger LDL particles. 

 This broad range of lipid improvements with aleglitazar addresses the pattern of dyslipidaemia often found in patients with type 2 diabetes. This agent may therefore have beneficial cardiovascular as well as anti-inflammatory effects, and long-term use may delay the progression of CVD. Adverse events with aleglitazar were mild (increases in body weight, the number of patients with oedema) and no indications of CVD or hepatotoxicity with this dual agonist was observed.

 Whether these benefits will result in a reduction of cardiovascular events is under evaluation in the large phase III study ALECARDIO. This study will also address the safety and tolerability of aleglitazar with a special focus on common PPAR-*γ*-related side effects such as weight gain, fluid retention, and bone fractures.

### 3.6. New Omega-3 Fatty Acid Formulations

Two new formulations of omega-3 fatty acids may provide additional TG-lowering effects by reducing VLDL production and increasing their catabolism. AMR101, which contains ≥96% eicosapentaenoic acid (EPA), ethyl ester, and no docosahexaenoic acid (DHA), reduced TG (relative to placebo, at 4-g/day dose) by 33% in patients with hypertriglyceridaemia [135] and by 21.5% in patients with mixed dyslipidaemia also receiving statin [136], significantly reduced non-HDL-C, apoB and VLDL-C and did not increase LDL-C [135, 136]. The ongoing reduction of cardiovascular Events with EPA-Intervention Trial (REDUCE-IT) is a cardiovascular outcomes study of AMR101 4 g/day in approximately 8000 patients at high risk for CVD events (http://clinicaltrials.gov/ct2/show/NCT01492361). Another new omega-3 fatty acid formulation, an ultrapure mixture of free fatty acid forms of EPA and DHA that also provides better absorption than traditional omega-3 preparations, is in phase III clinical trials in patients with hypertriglyceridaemia (EVOLVE; http://clinicaltrials.gov/ct2/show/NCT01242527) and added onto statin therapy in patients with mixed dyslipidaemia (ESPRIT; http://clinicaltrials.gov/ct2/show/NCT01408303).

### 3.7. Lipoprotein (a) Lowering Drugs

Lp(a) has been considered a cardiovascular risk factor for a long time and during the last few years, major advances have been achieved in understanding the causal role of elevated Lp(a) in premature CVD [137]. Although the benefits of lowering Lp(a) *per se* are still not demonstrated, a number of clinical and experimental studies, including mendelian randomization studies, indicate that this lipoprotein is causal in CVD [138, 139]. Whether this occurs by proatherogenic mechanisms, enhancing coagulation, or both remains to be addressed. Compared with LDL, Lp(a) is relatively refractory to both lifestyle and drug intervention. The data on the effects of statins and fibrates on Lp(a) are limited and highly variable. Overall, statins have, however, been shown to consistently and modestly decrease elevated Lp(a) in patients with heterozygous familial hypercholesterolaemia. Niacin reduces Lp(a) levels by up to 40% in a dose-dependent manner and in addition exerts other potential beneficial effects by reducing LDL-C, total cholesterol, TG, and remnant cholesterol and by raising HDL-C [137]. Niacin (1–3 g/day) reduces major coronary events, stroke, and any cardiovascular event by 25–27%. However, controlled-intervention trials with selective reduction in plasma Lp(a) levels aimed to reduce CVD are urgently needed; selective Lp(a) apheresis may represent such an approach [137]. Other agents reported to decrease Lp(a) to a minor degree (10%) include aspirin, L-carnitine, ascorbic acid combined with L-lysine, calcium antagonists, angiotensin-converting enzyme inhibitors, androgens, oestrogen and its replacements (e.g., tibolone), and antiestrogens (e.g., tamoxifen), while the development of a thyroxine derivative such as eprotirome, although effective in reducing Lp(a), was halted because of long-term cartilage damage in preclinical studies. The ongoing HPS2-THRIVE trials and the data on the Lp(a) from AIMHIGH will provide further information, although the niacin employed in these trials is not selective for Lp(a) lowering as noted above. 

More recently early preclinical studies suggest that targeting liver expression of apo(a) with ASOs directed to KIV-2 repeats—which are expressed in multiple copies in the human apo(a) gene—may provide a highly effective approach to lower elevated Lp(a) levels in humans. The development of such ASOs to lower Lp(a) levels might then allow clinical tests of the importance of lowering Lp(a) levels for the therapy and prevention of CVD.

It is clear that more detailed studies of the metabolism of Lp(a) are required to aid in the design and development of selective and potent therapies for lowering Lp(a) [137]. Given the critical role of Lp(a) synthesis in determining the plasma concentration of Lp(a), targeting either the synthesis of apo(a) and/or the formation of Lp(a) would appear worthwhile. ASOs, PCSK9 inhibitors, apoB synthesis inhibitors, and CETP inhibitors all affect Lp(a) plasma levels and may hold promise for the future.

## 4. Conclusion

Although statins provide effective and substantial reductions in LDL-C, non-HDL-C, and apoB, as well as other drugs provide beneficial effects on other lipids and lipoproteins, many patients do not achieve the recommended goals despite maximal therapy, and some patients cannot tolerate high-dose statin therapy. Available agents combined with statins can provide additional benefit on LDL-C reduction, and agents in development may increase therapeutic options. Genetic insights into mechanisms underlying regulation of LDL-C levels have expanded potential targets of drug therapy and led to the development of novel agents that are still undergoing testing to determine efficacy and safety. Alternative targets such as triglycerides, HDL, and Lp(a) also require attention; however, the available data are still not conclusive. Drugs increasing HDL may not be all alike and require adequate scrutiny of the mechanisms involved. Drugs increasing apoA-I availability may represent the best approach. Lp(a) also represents an attractive target; however, it will be difficult to address, with currently available intervention, whether decreasing Lp(a) provides a reduction in cardiovascular risk. The most promising approaches such as apoB synthesis inhibitors or PCSK9 inhibitors all decrease LDL as well. Until we have a better understanding of these issues, further LDL lowering in high-risk and very-high-risk individuals is the most sound clinical approach.

## Figures and Tables

**Table 1:** Established pharmacological agents for dyslipidaemia management and their effects on lipid fractions.

Agent	Lipid fraction (%)		
	LDL-C	HDL-C	TG
Bile acid sequestrants	15–30↓	3–5↑	No change
Ezetimibe	18–20↓	1–4↑	8↓
Statins	18–55↓	5–15↑	7–30↓
Fibrates	5–20↓	10–20↑	20–50↓
Nicotinic acid	5–25↓	15–35↑	20–50↓
Ezetimibe + statin	+15–20↓ versus statin alone		
Fibrate + statin	+5↓ versus statin alone	+5↑ versus statin alone	
Nicotinic acid + statin	+8–31↓ versus statin alone	+17–32↑ versus statin alone	+24–27↓ versus statin alone
